# Recruitment of a probability-based general population health panel for public health research in Germany: the panel ‘Health in Germany’

**DOI:** 10.1186/s12874-025-02746-4

**Published:** 2025-12-23

**Authors:** Johannes Lemcke, Ilter Öztürk, Stefan Damerow, Tobias Heller, Sabine Born, Matthias Wetzstein, Jennifer Allen, Patrick Schmich

**Affiliations:** Department 2 Epidemiology and Health Monitoring, Robert Koch Institute, Gerichtstraße 27, Berlin, 13347 Germany

**Keywords:** Mixed-mode survey, Probability-sampling, Selection effects, Study infrastructure, Incentives, Panel study, Panel recruitment study

## Abstract

**Background:**

This report presents the study design and recruitment outcomes for the ‘Health in Germany’ panel, a long-term population-based health survey infrastructure developed by the Robert Koch Institute. The initial recruitment was conducted using a stratified random sample of the German population and a mixed-mode approach combining web-based and paper questionnaires. We examine participation rates across demographic subgroups, assess sample composition, and analyze potential selection effects.

**Method:**

The panel recruitment survey for the ‘Health in Germany’ panel utilized the residents’ registration offices as the sampling frame. A two-stage stratified (cluster) sample was drawn from 359 primary sampling units across Germany. A mixed-mode approach was employed, offering both online and paper survey-mode on the basis of age groups. The sequence of survey modes was differentiated by age groups based on information from the residents’ registration offices. For respondents aged 16–69 years, a sequential mixed-mode design, offering the online mode first and only with the second reminder the paper survey-mode (also called push-to-web strategy), was applied. For respondents aged 70 years and older a simultaneous mixed-mode design was used, offering the online and paper survey-mode with the invitation. Participants completed a first panel recruitment survey in which socio-demographic and health data were collected. Selection effects and sample composition were analyzed via logistic regression models and compared with official population data.

**Results:**

A total of 62,556 interviews were conducted, with 49,766 participants consenting to join the panel. After double opt-in registration process (for online participants), 47,863 remained. Response rates were higher among women and younger participants. Online participation predominated in the sequential design, whereas offline participation was more common in the simultaneous design. Logistic regression indicated higher participation among women and residents of smaller municipalities. Overall, the sample composition aligned broadly with population benchmarks, except for education and citizenship.

**Conclusions:**

The first recruitment study for the ‘Health in Germany’ panel established one of Europe’s largest population-based health panels through a mixed-mode design. Future expansions include regular health surveys, biometric measurements, complementary self-recruitment alongside the probability-based sample, and the development of a mobile survey app.

## Introduction and background

 Evidence-based estimations of the health situation of the population in Germany require reliable and valid data sources. The Robert Koch Institute (RKI) offers a centralized information service in the form of federal health reporting to retrieve and classify this health-relevant information [1]. The relevant data sources include primary data from surveys and examinations and secondary data in general (e.g., secondary data from health insurance funds).

In the past, the RKI has collected primary data in various study formats (examples here are previous monitoring studies, such as the Study on the Health of Children and Adolescents (KiGGS) [2], the Study on the Health of Adults in Germany (DEGS) [3] and the German Health Update (GEDA) [4]). The studies listed provide high-quality survey and examination data for Germany.

However, past experience – especially during the COVID-19 pandemic (with the exception of the GEDA study format [4] – has shown that these study designs are difficult to adapt to the ad hoc information needs of the scientific community, policymakers, and the informed public. The COVID-19 pandemic demonstrated more clearly than any previous global event how crucial it is to have continuous and, above all, rapidly available data on population health. In the absence of such an infrastructure, the RKI had to generate further data on the incidence of infection using other surveys. For example, the sero-prevalence in the population was estimated in cooperation with the DIW’s Socio-Economic Panel (SOEP) [5]. These data are important for estimating the incidence of infection and the possible resulting burden on the healthcare system. The RKI will meet these needs, among others, with the panel infrastructure to be described here [6]. This will create the largest public health panel in Germany on the basis of a probability sample. Panel infrastructures that also rely primarily on the online survey mode (without systematically excluding the offline population) also offer an enormous advantage in terms of speed and cost. A significant advantage of the panel approach is that it allows changes in individuals to be recorded over time and enables longitudinal analyses. Such population-wide longitudinal studies are highly important for epidemiology and public health [7]. Under certain conditions, panel studies also allow causal effects to be estimated. This is particularly interesting for epidemiological studies that aim to evaluate national prevention measures (e.g. smoking prevention policies) at an individual level [8]. Panel studies also enable researchers to address selection effects and thus nonresponse bias resulting from attrition in a more complex way by using longitudinal weights. These weights are based on richer recruitment survey data than design weights, which rely only on register information. This enhances the accuracy and validity of the weights. Furthermore, a panel design allows for repeated cross-sectional surveys to analyze time series and trends, provided that the panel is regularly refreshed, appropriate cross-sectional weights are calculated, and panel attrition is closely monitored. The panel infrastructure will continuously collect three key types of data and make them available in a timely manner: survey data, measurement data (e.g., height and weight collected in person), and laboratory data (e.g., from dried blood analyses) [6]. ‘Health in Germany’ is being developed in the context of other large, well-established (mixed-mode) panel infrastructures in the (social) sciences. The SOEP (Socio-Economic Panel at the German Institute for Economic Research, DIW) and the GESIS Panel (GESIS - Leibniz Institute for the Social Sciences) in Germany are significant and influential examples of such infrastructures. Internationally, the LISS panel (Longitudinal Internet Studies for the Social Sciences) in the Netherlands is recognised as a pioneer in this mixed-mode probability-based panel context. In the USA, the Understanding America Study has established another important, exemplary panel infrastructure of this kind. These implementations demonstrate the feasibility and benefits of such infrastructures for science [9–14]. Like the above-mentioned panels, ‘Health in Germany’ is planned as a self-administered mixed-mode panel (online and postal survey). Particularly in the last 10 years, many large survey infrastructures have switched from interviewer-administered to self-administered surveys, a trend that accelerated during the first waves of the COVID-19 pandemic due to contact restrictions [15].

This field report contributes to the growing body of literature on mixed-mode survey designs and population-based health panels. Within the public health field, see [16] and [17] for push-to-web recruitment and [18] for a general discussion of the study design. Here, we provide insights into the feasibility and practical implementation of a push-to-web strategy in large-scale health surveys, particularly in the context of Germany. Furhtermore we investigate the nonresponse bias in the study, which can arises from systematic selection effects. Compared to a cross-sectional study, selection into a panel is an additional selection stage and therefore also a potential source of nonresponse. In the analysis of survey samples, the presence of selection effects is of particular concern, given these effects represent differences between respondents and the target population [19]. The significance of these factors lies in the potential for bias in estimation, particularly in cases where health outcomes are associated with the characteristics of underrepresented groups. Therefore, selection effects may be dependent on the specific characteristics of the study population. Previous studies have shown that participation in surveys, independent of survey mode, is significantly linked to socio-demographic factors. Older people within a certain age range, women, and individuals with a higher socio-economic status – reflected in income and education – are more likely to participate in many studies [20–23]. In addition, research in epidemiological methods has shown that health survey respondents often report better subjective health [24, 25], engage in healthier behaviors (e.g., lower alcohol consumption, more frequent physical activity, and less risky sexual behavior [20, 26, 27]), and indicate better mental health compared with non-participants [25, 28]. These results show that it is unrealistic to expect to detect no nonresponse bias. For this reason, it is important to determine and present the effect size. In thisfield report, we describe the study design, sampling and recruitment of the ‘Health in Germany’ panel [6]. We address the potential non-response bias in both the initial panel recruitment survey and the subsequent panel registration. Specifically, in this field report, we show the achieved outcome rates (also known as participation rates) differentiated by subgroups. Since survey outcome rates, such as the overall response rate, are a poor indicator for nonresponse bias alone [29], we additonally present sample composition in comparison with external benchmark data. Furthermore, data from the sampling frame and the panel recruitment survey are used to identify potential selection effects in the panel registration process. The field report will analyse the following questions.:

1. To what extent are there substantial differences in participation rates between subgroups (differentiated by information of the sampling frame e.g. age, sex)?
2. Can a systematic and substantial and statistically significant selection be observed when registering for the panel (regarding sociodemographic and public health related variables from the panel recruitment survey)?
3. How large are the differences between the sample composition in terms of socio-demographic characteristics and the external benchmark data (microcensus)?

## Methods and design

The recruitment study followed an age-differentiated mixed-mode approach [12, 30–32]. The following sections first discuss the sampling frame, the sampling procedure and the overall study design.

### Sampling frame

For the initial sampling in the panel’s first recruitment study, the population register maintained by the residents’ registration offices (in German: “Einwohnermeldeämter”(EMA), also known as the EMA sampling frame), which are organized at the local or municipal level, was chosen. This sampling frame offers several advantages over other established sampling frames in Germany. The use of official registers, such as residents’ registration offices, can lead to higher response rates (compared with telephone recruitment) and reduce nonresponse bias, especially education bias. This is evident when comparing the RKI study GEDA 2019/2020-EHIS, which used a telephone sample, with GEDA 2014/2015-EHIS, which used residents’ registration offices (EMA) [4, 33]. However, it is clear that the EMA sampling allows for postal, mixed-mode and face-to-face surveys, which generally have higher response rates than telephone surveys, which should be addressed as one limitation of this comparison. Another key advantage of this sampling frame is that potential nonresponse bias can be identified and corrected by weighting for variables such as age, sex, and region. The EMA sampling framework is ideal for self-administered panels, as participants do not have to switch from telephone recruitment to a self-administered mode, minimizing potential selection effects. However, a disadvantage of EMA recruitment is the possible cluster effect due to selection via sample points. To provide a concrete example, individuals within a given municipality may share socioeconomic or cultural characteristics (e.g., lower educational attainment, regional health behaviours), resulting in responses that show greater similarity among individuals within the same municipality than among individuals from different municipalities. This concept is commonly quantified by the intra-class correlation coefficient (ICC). It has been reported that ICCs for health-related outcomes across municipalities frequently range between 0.01 and 0.05. This has the potential to inflate standard errors by approximately 20–50% when cluster sizes are moderate (e.g., 20–30 respondents per municipality) [34, 35].

### Sample

The study employed a two-stage stratified cluster sampling design. In the first stage, 359 primary sampling units (PSUs, also referred to as sample points, SPs) were selected from municipalities across Germany (*N* = 10,789). This step was carried out in collaboration with GESIS Mannheim (Leibniz Institute for the Social Sciences). The allocation of SPs to each federal state was determined by the size of its population aged 16 and older relative to the total population of Germany in that age group, so that larger states received more SPs. To ensure that every federal state had a sufficient number of cases for reliable analysis, a minimum of 14 SPs per state was guaranteed by adjusting the calculated weights to this threshold. That was the case for nine of the 16 federal states. The final number of SPs per state was rounded using the Cox algorithm [36].

After the number of SPs had been determined for each federal state, the final selection of SPs within federal states took regional characteristics into account by subdividing each federal state according to the BIK classification, a commonly used regional standard in Germany for stratified sampling designs [37]. Strata weights were again calculated proportionally to the number of residents aged 16 and older within each state–BIK combination and were rounded using the Cox algorithm. Due to their large populations, several major cities were represented by multiple SPs.

In the second stage, address lists from the local residents’ registration offices of all municipalities included in each SP served as the final sampling frame. For SPs where selecting all addresses would have covered more than 70% of the eligible population, synthetic SPs were created by combining two or more similar municipalities. These synthetic SPs were identified using a k-nearest neighbor (kNN) algorithm based on the first six digits of the municipality code (in German: AGS - Amtlicher Gemeinde Schlüssel), municipality size, and BIK classification within the same district. Consequently, a single SP could comprise multiple municipalities.

From each selected SP, 600 addresses were initially drawn by the local residents’ registration offices according to sampling instructions provided by the RKI. From these, RKI researchers randomly selected 400 addresses for invitation, while the remaining 200 served as a reserve sample in case response rates were lower than expected. The selection was carried out using age-stratified random sampling across the following age groups: 16–29, 30–39, 40–49, 50–59, 60–64, 65–79, 80–84, and 85+. The number of addresses per age group was determined based on the corresponding proportion within the SP and expected recruitment rate for the panel. The minimum target sample size for the first recruitment study was set at 30,000 panel participants. For a power estimate and the underlying assumption, see [6]. The figure of 30,000 panel participants was derived from the 400 addresses per each of the 359 SPs, combined with age-specific assumptions on panel recruitment rates ranging between 12% and 22%, leading to 84 participants per SP.

The sampling unit was the individual (aged 16 years or older). Within each municipality, all eligible individuals had equal selection probability except for the age-based stratification in the final address draw.

As a special measure, the sample size per sample point was increased (oversampling) in the federal states of Berlin and Schleswig-Holstein to achieve larger subsamples within these states, thereby enabling more fine-grained analyses at the state level.

Before creating the final gross sample, all address data from the registration offices were processed. The term ‘gross sample’ refers to all addresses that were contacted for the study, sometimes also described as the ‘invited sample’. Quality checks were carried out by the RKI to account for the varying quality of address information provided by the registration offices. Addresses were standardized for relevant variables, and duplicates or invalid cases were removed. To detect these issues more comprehensively, an external service provider’s tool (ADRESSFACTORY by Deutsche Post) was used. A total of 14,732 addresses were identified and subsequently removed based on data from ADRESSFACTORY. The majority were addresses belonging to businesses or addresses that were no longer current, for example because inhabitants had moved or died. A few hundred addresses of people who had moved inside the same SP were also updated. The data quality was checked for completeness, plausibility and consistency among the relevant variables (names, birthdates, addresses, nationality codes etc.). In case of data shortcomings, usually indicating a non-random or faulty sampling process by the registration offices, the affected offices were contacted again to collect a second sample that met the required standards. Finally, the gross sample was divided into three batches to optimize the mailing process and allow a staged start of data collection.

After the final processing of the addresses, the gross sample comprised 166,843 addresses.

### Study design

**Fig. 1:**
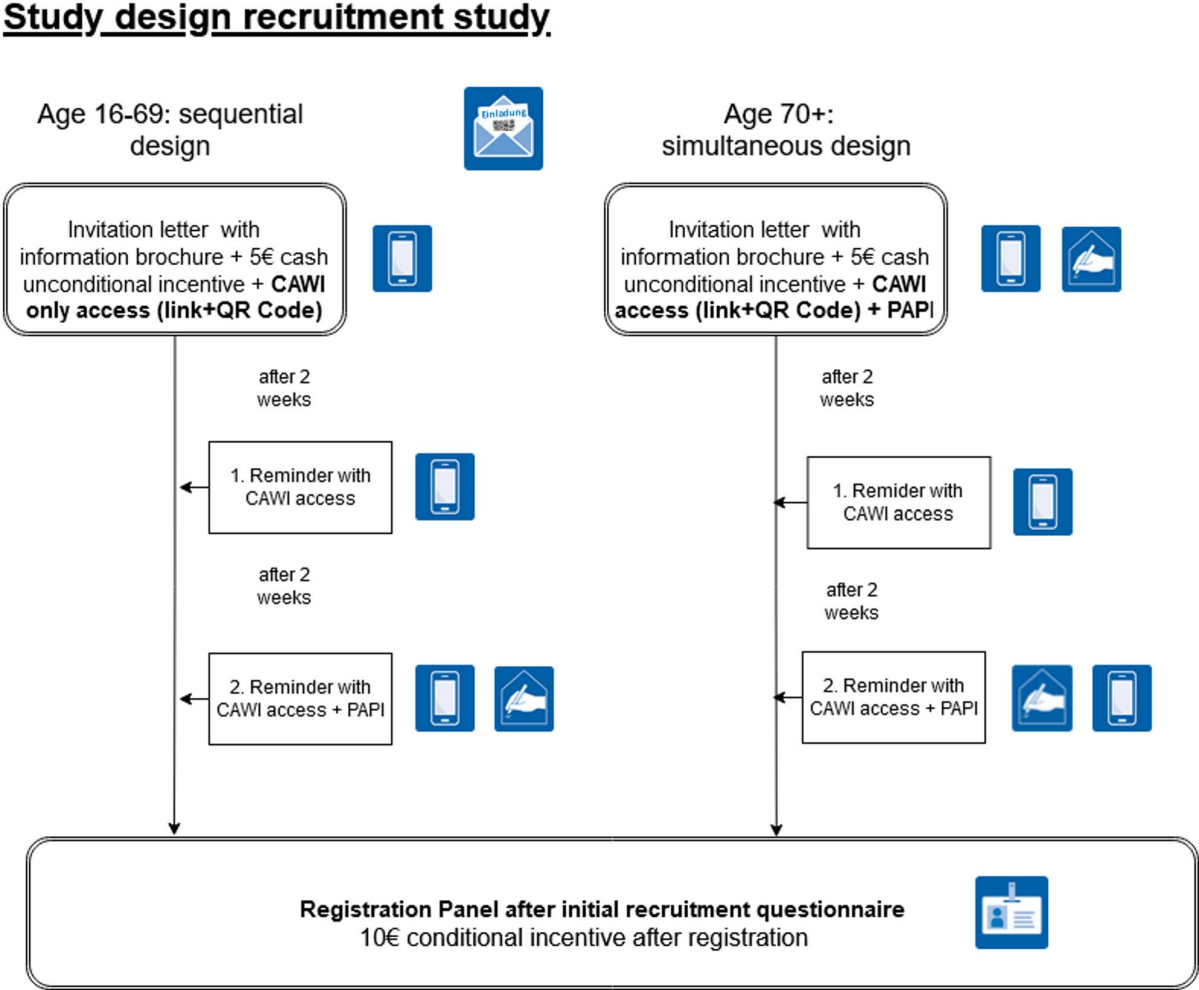
Simplified mixed-mode study design of the panel panel recruitment survey

As already mentioned, the recruitment study was designed as mixed-mode study. The sequence of survey modes offered was differentiated according to age groups, based on information available from the residents’ registration offices selection framework:

- Age group 16–69 years: Sequential mixed-mode design (push-to-web strategy) in the order shown in Fig. 1 (CAWI - Computer Assisted Web Interview; PAPI - Paper and Pencil Interview).
- Age group 70 + years: Simultaneous mixed-mode design.

Initially, all persons received a postal invitation letter containing a cover letter, an information brochure and an unconditional €5 cash incentive. Upon successful registration for the panel, participants received an additional €10 cash incentive (conditional incentive). This approach to incentivization was based on existing research and on an incentive experiment conducted in the run-in phase of the panel recruitment survey (publication still pending) [38]. Importantly, unconditional cash mailing is common in postal mixed-mode studies both internationally and in German-speaking countries, and represents an effective measure for increasing response rates in the recruitment of panel studies [9–11, 30]. People aged 16–69 years received only online access details (link and QR code) for the CAWI mode, while those aged 70 and over also received a paper questionnaire (PAPI mode) due to lower internet usage in this age group [39]. To begin the online survey, participants had to enter the integrated unique access code, which functioned as a password. Two weeks after the invitation letter, non-respondents were sent a reminder letter containing the CAWI access data but no paper questionnaire. A further two weeks later, a second reminder was sent to all remaining non-respondents, regardless of age group, and included a paper questionnaire as well as the option to respond via online survey.

A company [40] was commissioned by the Robert Koch Institute to manage the fieldwork of the mixed-mode survey and to provide active field supervision for subsequent survey waves planned in 2024. In this role, infas was responsible for the following tasks: printing, dispatching and managing the return of the written questionnaires; setting up a study hotline; and providing support to participants. The RKI provided the online survey software and the panel website [41, 42] and was also in charge of the communication concept. Additionally, the RKI and infas worked closely together to ensure data subject rights.

The survey was launched on 8 January 2024 and ended on 29 May 2024. The first, 10% of addresses were contacted during the run-in phase (batch 0). During this phase, the technical processes were checked, an incentive experiment was carried out, and the response management was fine-tuned with the external service provider. The field phase was completed on 29 May 2024. Prior to this, the invitation to batch 1 (50% of the addresses) was sent on 5 March 2024, and the invitation to batch 2 (the last 40% of the addresses) was sent on 20 March 2024. The first online interview of batch 0 was conducted on 9 January 2024, and the first completed paper questionnaires were recorded on 15 January 2024. The field was completed for all the batches together. The last written responses were included in the data collection on 29 May 2024. The information from paper questionnaires received by infas after this date was no longer recorded.

## Panel recruitment survey

Before the participants could register for the panel, they were invited to take part in an initial panel recruitment survey. This first survey collected sociodemographic and health-related data from the participants. The following question items were included:

- Basic socio-demographic information: age, sex at birth, education, household size, and citizenship.
- Health data: self-reported height and weight (used to calculate BMI).
- Chronic conditions: presence of long-term health problems (Yes/No).
- Self-rated health: overall and mental health assessed on standard scales.
- Health behaviour and lifestyle: smoking status, frequency of physical activity, and dietary habits (red meat and sausage consumption).
- Health awareness: degree of attention paid to personal health (from “not at all” to “very strongly”).
- Well-being: life satisfaction measured on a 11-point scale.
- Climate change: perceived health risks from climate change, categorized by level of concern.
- Healthcare access: whether participants experienced long waiting times for medical appointments in the past year.

This panel recruitment survey had two primary objectives. Firstly, the respondents should gain an understanding of the topics they would be surveyed on in the panel in the future. In addition, this first survey served to record important variables that would then allow selection effects to be estimated and weighting factors to be calculated.

The questionnaire was only available in German. The anticipated questionnaire length for the online questionnaire was 10 min. The actual online questionnaire length was around 6 min (Mean: 7.5 min; Median: 5.9 min; 10% percentile: 3.5 min; 90% percentile: 11.9 min; SD: 6.9 min). The paper questionnaire consisted of six pages.

After completing the panel recruitment survey, online participants who agreed to be recontacted again were redirected to the registration page of the panel portal (https://gesundheit-in-deutschland.de/de). There, they entered their name, email address, postal address, date of birth, sex, and optionally, a phone number and create an account. They then received an automated verification e-mail containing a confirmation link, which led to the final registration in the panel (double opt-in procedure). From this point onward, participants who registered online were considered active panelists and became available for regular panel operations. Participants who registered for the panel via the PAPI mode went through a slightly modified registration process. The relevant registration parameters (name, sex, date of birth, and postal address) were recorded on the basis of the returned declaration of consent for panel participation. Once registered, it was not possible for participants to change their chosen survey mode for future waves.

Throughout the 5-month survey period, a hotline was available for all invited persons, operated during regular business hours by trained staff of the service provider. In addition, study websites hosted by the service provider and on rki.de provided further information about the study’s content and procedures. The RKI also issued a press release at the start of the main survey phase and marketed the study via the RKI’s social media channels.

### Calculation of participation rates

Participation rates were calculated according to the standards of the American Association of Public Opinion Research (AAPOR) [43]. The response rates RR1 and RR2, as well as the web rate, panel consent rate and recruitment rate (RECR), are reported in Table 1 (the exact distribution of the disposition codes can be found in Appendix Table 5). For the recruitment study, the AAPOR disposition codes were adapted to the German context following Stadtmüller et al. [44]. RR1, also known as the Minimum Response Rate, is defined as the number of complete interviews divided by the number of interviews plus the number of non-interviews plus all cases of unknown eligibility. RR2 follows the same logic but additionally includes partial interviews, defined as cases where consent was given and at least the first two items were completed. The web rate indicates the ratio between online and offline participants in the panel recruitment survey. The panel consent rate represents the proportion of respondents who fully or partially completed the recruitment interview and additionally consented to be recontacted by the RKI in the future (regardless of actual registration, for CAWI participants, this includes participants who have not fully completed the double opt-in registration process.). The recruitment rate (RECR) indicates the proportion of invited persons who participated in the recruitment survey, provided panel consent, and successfully completed the double opt-in registration process (for CAWI participants only), thus formally becoming active panel members. Response and recruitment rates were further analyzed by age group, sex and study design (Tables 2 and 3).

### Case matching and quality control

The outcome rates reported are based on the unadjusted data set, meaning that no case matching was performed. The EMA sampling frame allows basic verification of the participants on the basis of year of birth, month of birth, and sex at birth recorded in the selection frame compared with the self-reported information provided in the questionnaire. This verification process was performed in an initial case matching. If the three variables matched, the questionnaire was processed further. If discrepancies were found, the following procedures were applied to resolve them:

- Sex at birth: If the only discrepancy was in the recorded sex at birth, it was corrected according to the information provided in the questionnaire. If additional variables also deviated, the case was excluded.
- Month of birth: If the month of birth deviated by six months or less, the value was corrected based on the questionnaire. If it deviated by more than six months, the case was excluded. If the month of birth was missing in the EMA data, the tolerance limit was increased to twelve months.
- Year of birth: If there was a discrepancy in the year of birth, the case in question was excluded.

On the basis of the case comparison, the information that the participants provided in the questionnaire was also compared with the information they provided when registering for the panel. If discrepancies were identified in this further step (analogous to the rules described above), they were also excluded.

### Double realizations

Due to the mixed-mode study design, some participants completed the survey in more than one mode (PAPI and CAWI). This can occur because participation speeds differ across modes (e.g., returning PAPI documents takes longer than completing CAWI). The following rules were applied to handle such cases:

- The questionnaire with the higher degree of completion is retained; the other is marked.
- If completion is equal, the first completed questionnaire is retained; the other is marked.
- If completion and date are identical, the CAWI questionnaire is retained; the PAPI questionnaire is marked.

This procedure also applies to assigning registered panelists to survey modes for the final deployment sample. Consequently, the mode allocation in the deployment sample used for invitations in the first and subsequent panel waves may differ slightly from the original gross sample.

### Analysis of selection effects and sample composition

To analyze selection effects, logistic regression models are estimated for participation in the recruitment study and the willingness to register for the panel. As independent variables, information from the gross sample, which is available for all contacted individuals from the registration offices, is used:

- Age: Calculated from the registered birthdate and categorized into age groups (16–29 years, 30–39 years, 40–49 years, 50–59 years, 60–69 years, 70 + years).
- Sex: Officially recorded sex at birth (male, female).
- Region: Based on the resident’s federal state, regions are categorized as follows:
  - ◦ Northeast: Berlin, Brandenburg, Mecklenburg-Western Pomerania
  - ◦ Northwest: Schleswig-Holstein, Hamburg, Lower Saxony, Bremen
  - ◦ Central East: Saxony, Saxony-Anhalt, Thuringia.
  - ◦ South: Baden-Württemberg, Bavaria.
- BIK classification: Municipalities in Germany are classified based on their size and commuter flows [45]. The official ten categories are summarized as follows:
  - ◦ < 20,000 inhabitants.
  - ◦ 20,000 to < 50,000 inhabitants or surroundings of a city with 50,000 to < 500,000 inhabitants.
  - ◦ Core city with 50,000 to < 500,000 inhabitants or surroundings of a city with 500,000 + inhabitants.
  - ◦ Core city with 500,000 + inhabitants.

We used data from the panel recruitment survey to assess selection bias that may have occurred during the panel registration process, using additional sociodemographic and health-related indicators for illustration. To further investigate this, a logistic regression was conducted on the sample of participants from the panel recruitment study, with registered for the panel (Yes/No) as the dependent variable. This model included the variables from the gross sample selection analysis, supplemented by the following independent variables:

- Education: Categorized as low, medium, high according to Casmin classification [46].
- Household size: Single and multi-person household.
- German: self-reported information on holding German citizenship (Yes/No).
- BMI: Body mass index (BMI) was calculated from self-reported weight (kg) and height (m) as BMI = weight in kg/(height in m)² and categorized as follows:
  - ◦ Underweight (BMI < 18.5).
  - ◦ Normal weight (18.5 < = BMI < 25).
  - ◦ Overweight (25 < = BMI < 30).
  - ◦ Obesity (BMI > = 30).
- Chronic disease: Self-reported chronic disease (Yes/No).
- Self-rated health: Categories from “very good” to “very bad”, grouped into very good/good/fair vs. bad/very bad.
- Self-rated mental health: Categories from “Excellent” to “poor”, grouped into very Excellent/very good/good vs. fair/poor.
- Paying attention to health: Categories from “not at all” to “very strong”, grouped into not at all/less strong/moderate vs. strong/very strong.
- Satisfaction: Life in general: Scale from 1 (Completely dissatisfied) to 10 (Completely satisfied) categorized as 1 to 3, 4 to 7, and 8 to 10.
- Red meat: Consumption of red meat in categorized quantities: Daily or several times a day, 4 to 6 times per week, 1 to 3 times per week, less than once per week, never.
- Sausage products: Consumption of red meat in categorized quantities: Daily or several times a day, 4 to 6 times per week, 1 to 3 times per week, less than once per week, never.
- Smoking: Self-reported information on daily smoking, occasional smoking or non-smoker.
- Sport: Weekly duration of sporting activity during the past three months, categorized as follows: No sporting activity, less than 1 h per week, regularly 1 to less than 2 h per week, regularly 2 to less than 4 h per week, and regularly 4 or more hours per week.
- Subjective health risk due to climate change: Scale from 1 (Not at all) to 10 (Very much) categorized as 1 to 3, 4 to 7, and 8 to 10.
- Waited for appointment: Waited for medical appointment in the last 12 months.

The models were estimated in R (version 4.3.0) [47] using svyglmfrom the survey [48] package accounting for the sampling design and inverse probability weights. A statistically significant effect was assumed if the calculated p value is < 0.05. To illustrate the results, average marginal effects (AME) were calculated using the R-package margins (version 0.3.28) [49] and visualized with ggplot2 (version 3.5.1) [50]. The AME indicates “by how many percentage points the probability of the event of interest changes in the mean of all (group-specific) observations if the explanatory variable in question increases (marginally) by one unit” [51, 52]. In the case of categorical variables, the AME indicate by how many percentage points the average probability of the event of interest (in this case either participation in the panel recruitment survey or panel registration) in the group under consideration (e.g.the oldest age group, 70 + yrs.) differs from the probability in the respective reference group (e.g. youngest age group, 16–29 yrs.).

The sample composition was evaluated by comparing proportions of selected variables with benchmark figures at different stages of the recruitment process: gross sample, participants in the recruitment study, and the sample of registered panelists. In addition to the raw proportions, we calculated weighted proportions for both the recruitment study sample and the registered panelist sample. These weighted proportions accounted for the sampling design and inverse probability weights. As benchmarks, distributions from official statistics (Population figures (as of December 31, 2023) [53] and Microcensus 2021 [54]) were used. This allowed for an assessment of the extent to which the sample composition deviated from the actual composition of the target population. Proportions were calculated for the variables from information of the gross sample that were used in the selection analysis except that the federal state was used instead of the region. Additionally, proportions for education (Casmin classification: low, medium, high [55]), German citizenship and household size (single-person household, multi-person household) were derived for the samples of the recruitment study and the sample of registered panel participants, as this information was not available for the gross sample.

## Results

### Outcome rates

**Fig. 2:**
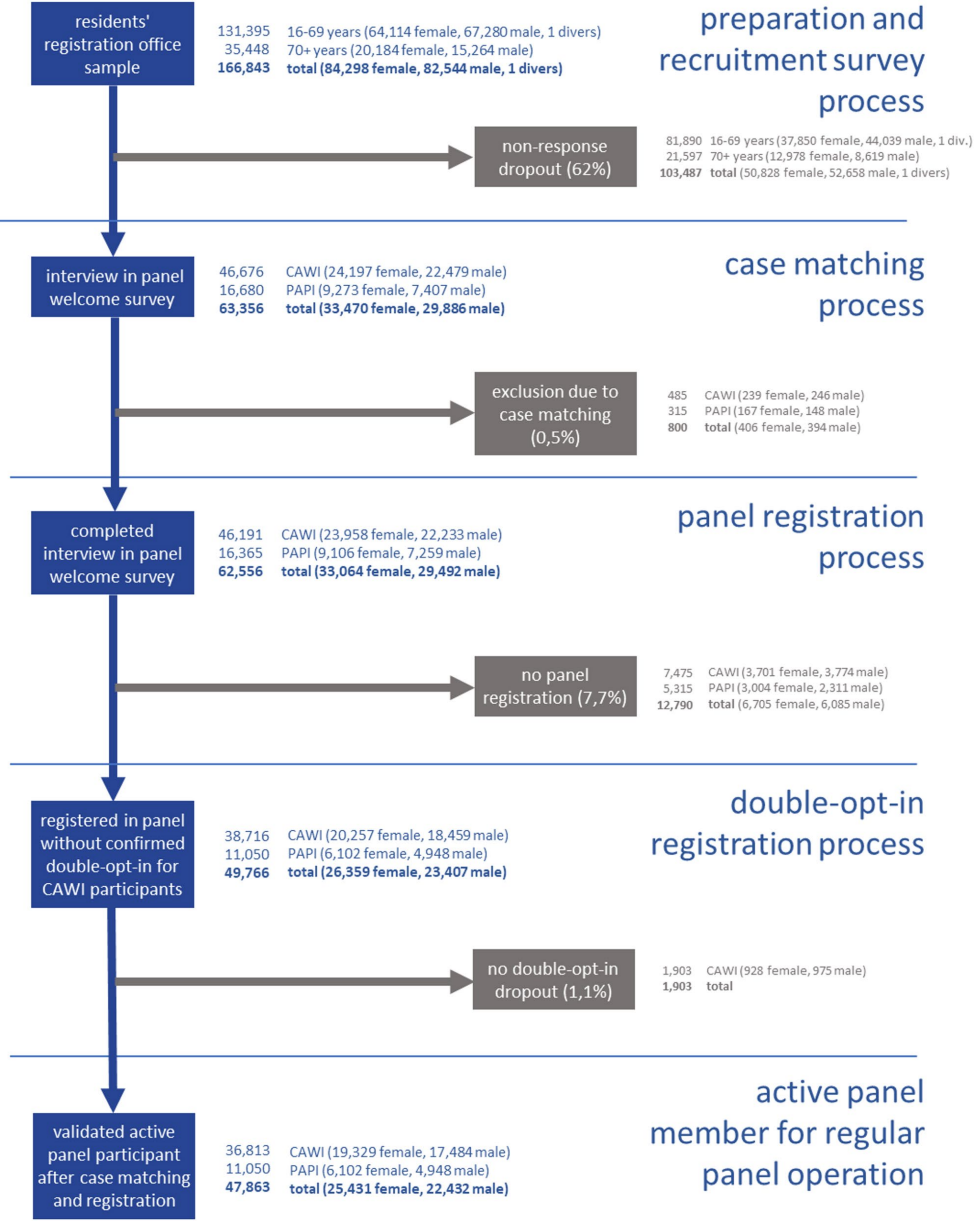
Selection levels of the panel recruitment survey, panel registration and panel validation; percentages represent the relative share of the gross sample

Figure 2 below shows all the selection stages of the panel recruitment process. A total of 62,556 complete recruitment interviews were conducted.

A total of 49,766 participants indicated their willingness to be recontacted (`panel consent’) and registered for the panel. These included 1,903 CAWI participants who did not click the confirmation link sent to them by email after registration and therefore could not be counted as active panelists, resulting in a total of 47,863 active (double-opt-in confirmed) panelists. In total, 2,726 double realizations were identified. The majority (86.0%) occurred once in CAWI and once in PAPI, while 13.4% were realized twice in PAPI and 0.6% once in CAWI and twice in PAPI. As described above, these cases were assigned to a mode according to the documented rules, resulting in a single valid interview or registered panel case.

Table 1 Shows the summary of outcome rates in the panel panel recruitment survey (for a detailed presentation of the response rate for the panel recruitment survey, see Appendix Table 5). The overall response rate (RR1) was 37.5%, with women (39.2%) demonstrating a higher level of participation than men did (35.7%), as shown in Table 2, differentiated by age group, sex, BIK classification and region group. An important result can be seen when the response rates are differentiated by age. The 16–19 years (44.0%), showed the highest response rates, whereas those aged 20–29 years presented the lowest response rate (34.7%). The middle age groups 30–39 years (35.6%) and 40–49 years (35.6%) also had slightly lower response rates compared to the older age groups, such as the 60–69 years (40.3%) age group. Differentiating the age groups by sex Shows that women almost consistently show a higher response rate. For instance, among those aged 16–19 years, the response rate for women was 48.8%, substantially exceeding the 39.9% reported for men in the same category. In contrast, there is a reversal of this trend in the older age groups. Men aged 70 + have the highest response rate within their sex group (42.9%), in contrast to women in the same age group (35.0%). Regarding the BIK classification, overall, the group residing in areas with 20,000 to less than 50,000 inhabitants or surroundings of a City with 50,000 to less than 500,000 inhabitants exhibited the highest response rate (38.7%), while the group from the core City with 500,000 + inhabitants showed the lowest response rate (36.4%). This pattern was consistent across both sex groups, with women demonstrating higher response rates than men in every BIK classification category. Concerning response rates by region, participants from Northwest Germany displayed the highest overall response rate (39.4%), whereas participants from Northeast Germany had the lowest (35.7%). Notably, within the sex groups, the highest response rates was observed in the Northwest group. Again, women had higher response rates than men across all regional groups. Interestingly, within the male subgroup, participants from the Northeast showed the lowest response rate (33.6%), whereas among females, the lowest response rate was found in the central East group.

**Table 1:** Summary of outcome rates of the panel recruitment survey

**Outcome Rate**	Panel recruitment survey
AAPOR Response Rate 1 (RR1)	37.5%
AAPOR Response Rate 2 (RR2)	37.6%
Web Rate	73.9%
Panel Consent Rate	79.3%
Recruitment Rate (RECR)	28.7%

An analysis of the ratio between CAWI and PAPI participation (web rate) revealed that 73.9% of the interviews were conducted via CAWI, while 26.1% were conducted via PAPI. Furthermore, 88.4% of those who were invited with the approach of the sequential study design participated in CAWI mode, and 11.6% in PAPI mode. In addition, the analysis showed that among those who received an invitation in the simultaneous study design approach, 76.7% participated in PAPI mode, and 23.3% in CAWI mode. However, these rates are confounded with an age effect due to the distribution of the different sequence designs based on age.

**Table 2:** Panel recruitment survey AAPOR response rate 1 (RR1)

Attribute	Men		Woman		Total	
	*N* Respondents	Response Rate (%)	*N* Respondents	Response Rate (%)	*N* Respondents	Response Rate (%)
Total	29,492	35.7	33,064	39.2	62,556	37.5
16–19 years	1,475	39.9	1,556	48.8	3,031	44.0
20–29 years	3,867	30.5	4,456	39.5	8,323	34.7
30–39 years	4,370	32.4	4,900	39.2	9,270	35.6
40–49 years	3,350	31.6	3,974	39.8	7,324	35.6
50–59 years	5,096	34.6	5,997	41.2	11,093	37.9
60–69 years	4,806	39.8	5,126	40.8	9,932	40.3
70+ years	6,528	42.9	7,055	35.0	13,583	38.4
< 20,000 inhabitants	3,044	35.6	3,372	39.0	6,416	37.3
20,000 to < 50,000 inhabitants or surroundings of a city with 50,000 to < 500,000 inhabitants	9,880	37.1	10,997	40.3	20,877	38.7
Core city with 50,000 to < 500,000 inhabitants or surroundings of a city with 500,000 + inhabitants	7,210	35.7	8,226	39.1	15,436	37.5
Core city with 500,000 + inhabitants	9,358	34.4	10,469	38.4	19,827	36.4
Northeast	4,828	33.5	5,446	38.0	10,274	35.7
Northwest	6,890	37.4	8,066	41.2	14,956	39.4
Central East	3,057	35.2	3,349	37.8	6,406	36.5
Central West	8,252	35.5	9,220	38.5	17,472	37.0
South	6,465	36.5	6,983	39.8	13,448	38.1

The overall recruitment rate was 28.7% (shown in Table 1 and differentiated by age groups, sex and study design groups in Table 3). Considering the age groups, the highest recruitment rate was observed in the youngest age group (16–19 years; 34.1%), and the lowest in the oldest age group (70 + years; 26.1%). When looking at the sex groups, a slightly higher recruitment rate was observed among female participants (30.2%) than among male participants (27.2%). These differences between the sex groups are evident across all age groups, except in the 70 + years age group. Here, male participants (30.2%) had a significantly higher recruitment rate than female participants did (23.1%). Regarding the BIK classification, it was found that the group living in areas with 20,000 to less than 50,000 inhabitants or in the surroundings of a city with 50,000 to less than 500,000 inhabitants had the highest recruitment (29.2%), while the group with fewer than 20,000 inhabitants had the lowest (27.5%). This pattern was also observed within sex groups, with female participants consistently showing slightly higher recruitment rates than male participants across all BIK categories.

With respect to regional differences, participants from northwest Germany demonstrated the highest overall recruitment rate (30.4%). This trend was mirrored in the sexcomparison, where the highest recruitment rates within both sex groups were also found in the northwest region. The lowest overall rate was observed in the northeast of Germany (27.4%). Among male participants, the lowest rates were recorded in both the northeast and central east regions (25.4%), whereas within the female group, the lowest recruitment rate occured in the central east region (27.8%).

**Table 3:** Panel recruitment rate (RECR)

Attributes	Men		Woman		Total	
	*N* Validated	RECR (%)	*N* Validated	RECR (%)	*N* Validated	RECR (%)
Total	22,432	27.2	25,431	30.2	47,863	28.7
16–19 years	1,100	29.8	1,247	39.1	2,347	34.1
20–29 years	3,035	23.9	3,702	32.8	6,737	28.1
30–39 years	3,494	25.9	4,036	32.2	7,530	29.0
40–49 years	2,609	24.6	3,158	31.6	5,767	28.0
50–59 years	3,920	26.6	4,730	32.5	8,650	29.5
60–69 years	3,679	30.5	3,912	31.1	7,591	30.8
70+ years	4,595	30.2	4,646	23.1	9,241	26.1
< 20,000 inhabitants	2,233	26.1	2,503	28.9	4,736	27.5
20,000 to < 50,000 inhabitants or surroundings of a city with 50,000 to < 500,000 inhabitants	7,405	27.8	8,310	30.5	15,715	29.2
Core city with 50,000 to < 500,000 inhabitants or surroundings of a city with 500,000 + inhabitants	5,543	27.5	6,364	30.3	11,907	28.9
Core city with 500,000 + inhabitants	7,251	26.7	8,254	30.2	15,505	28.5
Northeast	3,657	25.4	4,232	29.5	7,889	27.4
Northwest	5,247	28.5	6,316	32.3	11,563	30.4
Central East	2,212	25.4	2,464	27.8	4,676	26.6
Central West	6,374	27.4	7,018	29.3	13,392	28.4
South	4,942	27.9	5,401	30.8	10,343	29.3

### Selection effects

#### Selection effects gross sample

**Fig. 3:**
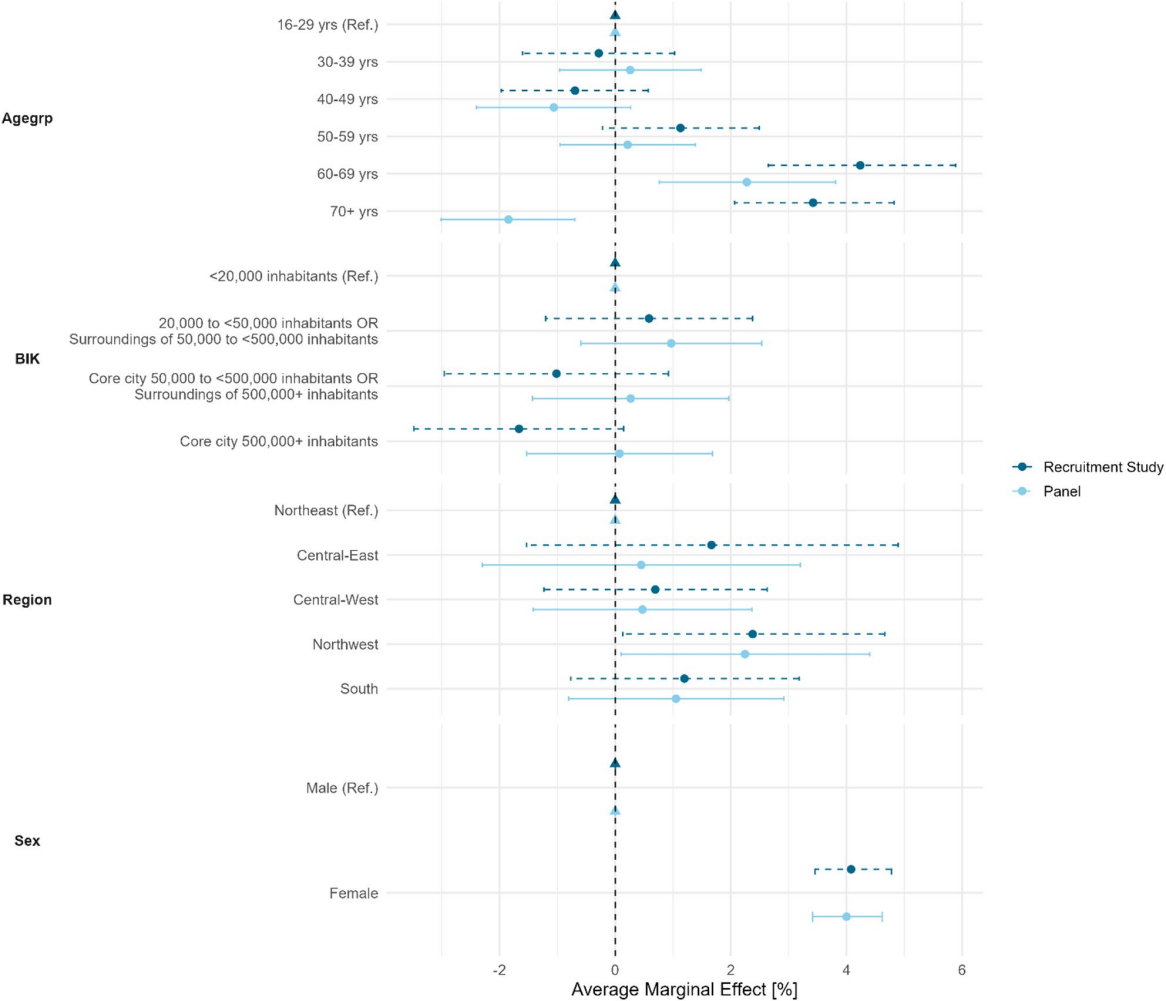
Average marginal effects with 95-% confidence intervall of logistic regression models of participation in the recruitment study [Yes/No] and panel registration [Yes/No]

Figure 3 shows the results of the logistic regression of participation in the recruitment study and registration for the panel with respect to the gross sample. The figure shows AME (the Appendix Table 6 & 7 contains the more comprehensive regression tables including Log-Odds and p-values) with 95% confidence interval.

The two models show similar patterns of participation probability. However, only a few variables have a statistically significant influence on participation. Compared to the youngest reference category, people in the 60–69 age group were statistical significant more likely to participate in both the panel recruitment survey and the panel registration. Opposing effects between the panel recruitment survey and panel registration can be observed for individuals aged 70+. The classification of the municipalities of the respondents via BIK classification had no effect on participation. In contrast, participants of the northwest region compard to the reference category, had a sligthliy higher likelihood to participate in both the panel recruitment survey and the panel registration. As already seen from the descriptive sub-group response rates, female respondents, compared to male respondents, showed a statistical significant higher likelihood to participate in the panel recruitment survey and the panel registration.

#### Selection effects health related variables form the panel recruitment survey

**Fig. 4:**
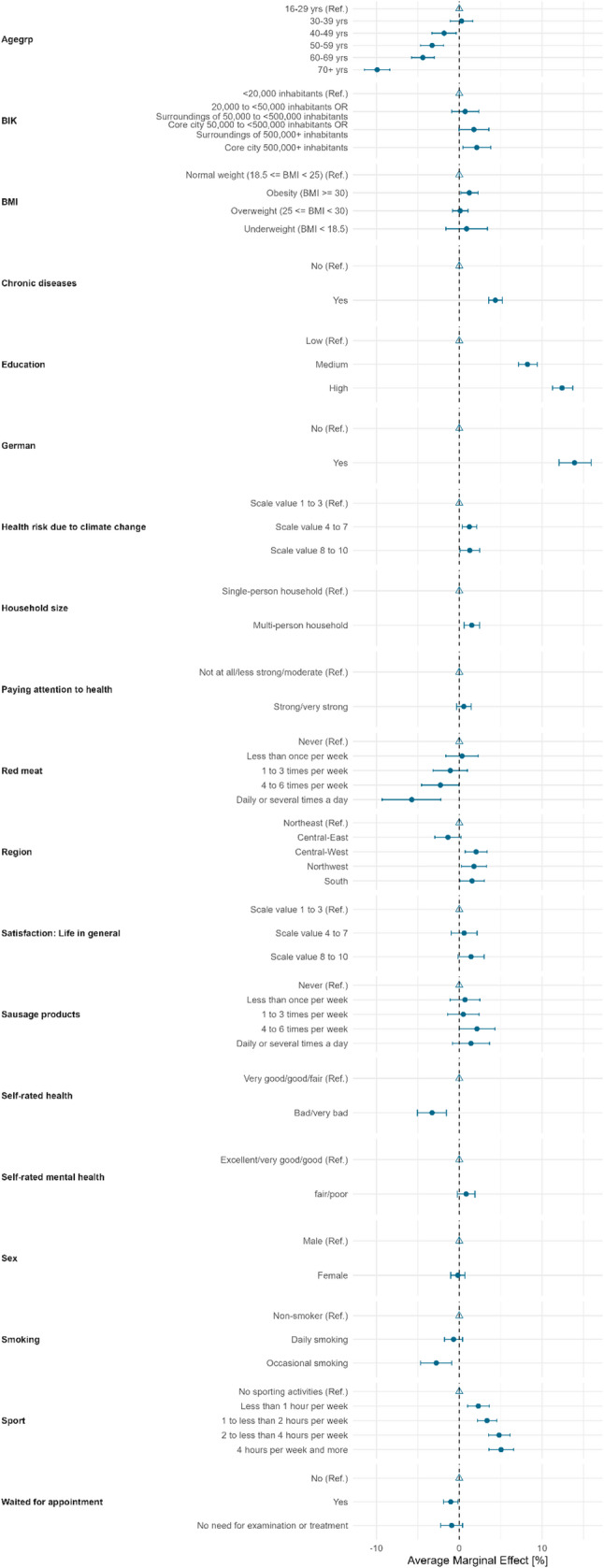
Average marginal effect with 95-% confidence intervall of logistic regression model of panel registration [Yes/No]

Figure 4 shows the selection analysis of the sociodemographic variables, extended by the health-related variables from the recruitment survey. Also shown are the AMEs (the Appendix Table 6 & 7 contains the more comprehensive regression tables including Log-Odds and p-values) with 95% confidence interval. The probability of registering for the panel after the recruitment survey was estimated. With regard to the socio-demographic variables, a similar pattern is shown, as in the selection analysis, which is based on information from the sampling frame. However, in this more comprehensive model, sex no longer has a statistically significant effect on the probability of panel registration. Education, in contrast, was positively related to participation. Respondents with medium and especially high educational attainment were more likely to register compard to respondents with low education. Moreover, respondents without German citizenship had a substantially and statistically significant reduced likelihood of registering. This is likely a result of the questionnaire being only available in German, which poses a significant barrier for some foreign residents with limited German language skills. Regional differences were small and statistically significant for the regions central-west, northwest and south showing a higher likelihood for panel recruitment of respondents living in these regions, compard to the reference category north-east. Household size was statistical significant associated with registration, showing that multi-person households had a slightly higer likelihood for panel registration. Perceived health risks due to climate change also showed robust effects. Respondets who had the highest and medium perceived threat had a higher statistical significant likelihood of become a panel member, when compared to the reference group. Having self-reported chronic conditions were statistical significant associated with a higher likelihood for registration, compard to the reference category of not having a chronic health condition. Respondents with a BMI equal or over 30 (definition of obesity) were statistically significantly more likely to register for the panel than those in the normal weight reference category. The same is shown fo respondents that had experienced longer waitings for doctors appointsments within the last year.

General self-rated health had a statistically significant effect on the likelihood of panel registration. Respondents rating their self-rated health as bad or very bad had a lower likelihood of registration, compared to respondents rating their self-rated health as very good, good, or fair. In contrast, self-rated mental health status showed no statistical significant effect. Health awareness (measured with the item “paying attention to one owns health”) had no effect on the likelihood of registration. Health behavior factors showed some relevant associations: occasional smokers were statistical significant less likely to register (compared to non-smokers), while individuals engaging in regular sports activities—particularly those exercising between two and four hours per week—demonstrated an increased probability of registration. In contrast, dietary habits such as sausage consumption were not related to panel registration. The daily consumption of red meat instead, is statistical significant negatively associated with panel registration. Other individual characteristics such as satisfaction with life in general did not show a clear association with registration.

### Sample composition

Table 4 shows the sample composition of selected variables for the gross sample, the different recruitment steps (recruitment study, panel registration) and benchmarks from official statistics.

For age, and BIK classification, only minor deviations can be observed between the gross sample, the unweighted recruitment study, and the unweighted sample of registered panelists. A slight shift is visible in the sex distribution, with women more frequently represented in the recruitment study (52.8%) and the panel (53.1%) compared to the gross sample (50.5%) and the benchmark (51.0%). Deviations from the benchmark distribution of federal states emerge in the unweighted samples, reflecting oversampling of smaller states as well as additional oversampling in Berlin and Schleswig-Holstein. After accounting for the sampling design (columns “RS Weighted” and “Panel Weighted”), these deviations largely disappear. For variables available only in the recruitment study and the panel substantial differences occur. In particular, respondents with high edudation level are overrepresented in the panel (31.3%) and recruitment study (29.1%) compared to the benchmark (19.9%), while lower levels of education are underrepresented (recruitment study: 23.3%, Panel: 20.1%, benchmark: 34.2%). German citizenship is reported more frequently among respondents of the recruitment study (93.6%) and in the panel (94.6%) than in the benchmark (85.0%). With respect to household size, the share of single-person households is lower among panelists (21.2%) than in the recruitment study (22.1%) and the benchmark (24.9%). Considering the sampling design reduces the proportion of single-person households by about 1% point, while the distributions of German citizenship and education remain essentially unchanged.

**Table 4:** Sample composition of the gross sample, recruitment study (RS), panel registration and population benchmark

Characteristic	Gross^1^ [%]	RS^1^ [%]	Panel^1^ [%]	RS^1^ Weighted^2^ [%]	Panel^1^ Weighted^2^ [%]	Benchmark^3^ [%]
Sex						
Male	49.5	47.2	46.9	47.3	47.1	49.0
Female	50.5	52.8	53.1	52.7	52.9	51.0
Age						
16–29 yrs	18.5	18.1	19.0	17.2	17.9	17.8
30–39 yrs	15.6	14.8	15.7	15.6	16.6	15.5
40–49 yrs	12.3	11.7	12.0	14.3	14.6	14.4
50–59 yrs	17.5	17.8	18.1	18.1	18.3	17.2
60–69 yrs	14.8	15.8	15.9	15.9	15.8	16.1
70 + yrs	21.2	21.9	19.3	18.9	16.7	19.0
BIK Classification						
< 20,000 inhabitants	10.3	10.3	9.9	10.5	10.2	10.8
20,000 to < 50,000 inhabitants OR Surroundings of 50,000 to < 500,000 inhabitants	32.3	33.4	32.8	35.6	35.1	34.6
Core city 50,000 to < 500,000 inhabitants OR Surroundings of 500,000 + inhabitants	24.7	24.7	24.9	26.2	26.5	26.8
Core city 500,000 + inhabitants	32.7	31.6	32.4	27.7	28.2	27.9
Federal State						
Schleswig-Holstein	8.8	9.7	9.8	3.5	3.6	3.5
Hamburg	3.4	3.5	3.7	2.2	2.3	2.2
Lower Saxony	7.1	7.6	7.5	9.7	9.6	9.6
Bremen	3.5	3.1	3.2	0.8	0.8	0.8
North Rhine-Westphalia	15.9	15.8	15.8	21.4	21.5	21.4
Hesse	5.4	5.4	5.3	7.5	7.5	7.6
Rhineland-Palatinate	3.6	3.5	3.6	4.9	5.1	4.9
Baden-Württemberg	9.7	9.6	9.7	13.3	13.4	13.3
Bavaria	11.5	11.9	11.9	15.8	15.8	15.8
Saarland	3.4	3.2	3.2	1.2	1.2	1.2
Berlin	10.3	9.8	10.1	4.4	4.6	4.5
Brandenburg	3.4	3.4	3.3	3.1	3.0	3.1
Mecklenburg-Western Pomerania	3.5	3.2	3.1	2.0	1.9	2.0
Saxony	3.7	3.8	3.7	4.9	4.7	4.9
Saxony-Anhalt	3.4	3.2	3.1	2.6	2.5	2.6
Thuringia	3.4	3.2	3.0	2.6	2.4	2.5
Education^4^						
Low		23.3	20.1	23.3	20.0	33.6
Medium		47.7	48.6	47.7	48.7	46.3
High		29.1	31.3	29.0	31.2	20.1
German Citizenship						
Yes		93.6	94.6	93.3	94.4	85.0
No		6.4	5.4	6.7	5.6	15.0
Household Size						
Single-person household		22.1	21.2	21.1	20.3	24.9
Multi-person household		77.9	78.8	78.9	79.7	75.1

^1^ Gross= Gross sample; RS = Recruitment Study; Panel = Registered Panelists

^2^ Weighting accounts for different selection probabilities from sampling design

^3^ Benchmarks calculated based on population figures [53]; Education and household size according to Microcensus [54]

^4 ^Casmin classification (Comparative Analyses of Social Mobility in Industrial Nations [46])

Table 8 in the Appendix reports Chi-square statistics, p-values, and Cramér’s V effect sizes for deviations of the sample distributions from the benchmark distributions for the characteristics presented in Table 4. Overall, while most Chi-square tests indicate statistically significant differences from the benchmark, the corresponding effect sizes are generally small, with values below 0.05 or ranging between 0.13 and 0.16. The largest deviations are observed for the federal state distribution in the unweighted samples (Cramér’s V ≈ 0.23–0.24), reflecting oversampling of smaller states and additional oversampling in Berlin and Schleswig-Holstein; these deviations are largely eliminated once the sampling design is taken into account.

In the age-stratified random sampling from the addresses provided by the residents’ registration offices, an error occurred in 12 municipalities. Some age cohorts were missing in the recruitment gross sample. This error predominantly affects small municipalities and has a negligible impact on overall analysis within federal states. An exception is Berlin, which, being a single municipality, is affected by the error in that there are no age cohorts from 85 years onward in the sample. An address update was arranged for the majority of municipalities, notably Berlin, to fill the gaps in age cohorts, alongside a new panel recruitment survey scheduled for 2025.

## Discussion

### Strengths

#### Participation rates and subgroup participation rates

In relation to the initial two research questions concerning participation rates and potential selection effects, several findings were observed.

The response rate of 37.6% achieved represents an above-average value compared with other surveys in Germany with a similar study design [12, 30, 56]. If one compares the recruitment rate (RECR) of 28.7%, which is crucial for the panel design, with other established panels, the above-average rate is also evident [57]. In their meta-analysis, Kocar and Kaczmirek reported a rate of approximately 16% (with a relatively high range) for a total of 23 comparable panel infrastructures [57]. This relatively high recruitment rate (within the german context of a mixed-mode survey) highlights the usefulness of the recruitment strategy employed in this study. Moreover, the panel achieved a comparable high consent rate of 76.9% for self-administered recruitment, which is particularly noteworthy considering that the majority of panel members participate via CAWI mode and have therefore completed a more complex double opt-in registration process. The advantage of this approach is that right at the start of the panel, there is a high data quality of the verified contact details, including a verified email address (compared to other recruitment methods where contact details might be self-reported without immediate verification). The use of a mixed-mode approach (PAPI and CAWI) has proven beneficial for reaching diverse population groups. However, it is important to note at this point that participation rates serve only as an indicator of data quality with respect to nonresponse and are not causally linked to it [58].

The targeted registration of 30,000 panelists was considerably exceeded, reaching over 47,000. The larger sample size will enable more in-depth analyses with the panel data than originally anticipated in the initial sample size and power calculations. A potential explanation for the higher response rate, despite the decline in response rates observed over the preceding two decades [59, 60], may be attributed to the impact of the ongoing pandemic in 2024. It is evident that the RKI has gained considerable visibility as a central public health institute in Germany. This could also have had an impact on the willingness to participate in the survey. There are other study results that have shown that the willingness to participate in surveys has generally increased (especially during the first phase of the pandemic and the first nation-wide lockdowns in spring 2020), regardless of the study topic (e.g. Becker et al. 2020 investigated this effect in a longitudinal panel [61]). However, such an increase in response rates was more likely to be seen in studies that did not have to switch modes. For example, studies that had to switch from face-to-face mode to self-administered mode showed a decline in the first phase of the pandemic (e.g. in UK Statistics [62]).

Regarding different subgroup participation rates different patterns were observed. Women were more likely to take part than men (response rate: 39.4% of women took part compared to 35.9% of men; recruitment rate: 30.2% of women took part compared to 27.2% of men). The youngest age group (16–19 years) had the highest rates of participation and recruitment (44.2% and 34.1% respectively). The oldest age group (70 + years) had the lowest overall recruitment rate (26.1%). A counterintuitive pattern was observed within this group: while women had higher rates in almost all other age groups, men over 70 showed a higher response rate (43.1%) and recruitment rate (30.2%) than women in the same age group (response rate: 35.2%; recruitment rate: 23.1%). One possible explanation for this difference could be a survivor effect: older men with lower levels of education may have a shorter life expectancy, while those who remain are likely to be in better health, potentially leading to a higher willingness to participate in health surveys. Studies suggest that although women generally live longer than men, they are more likely to experience physical limitations in old age [63], which could influence participation rates. It is also possible that socio-cultural or other unobserved factors influence the willingness of women in the 70 + age group to participate compared to men. This could be important factors in determining participation rates. This is indicated by the more comprehensive panel registration model, in which sex is no longer a significant factor in registration.

One of the major strengths of this new panel infrastructure is its capability to support longitudinal study designs and analyses, in addition to enabling high-quality cross-sectional studies. This feature is particularly valuable for research aiming to understand dynamic processes. That means e.g. changes in health status, health behaviors, risk factor exposure or other characteristics can be tracked on an individual-level, which is not possible with repeated cross-sectional studies. Furthermore, the high data quality achieved through the mentioned quality control measures, including thorough verification steps, ensures data integrity and reliability, which are crucial for longitudinal studies.

#### Selection effects and sample composition

The analysis of selection effects demonstrates that the sample of the panel recruitment survey and the sample of registered panelists show very similar effects when compared to the gross sample. This suggests that the registration process introduces only minor additional selection effects relative to the cross-sectional sample of the panel recruitment survey. Furthermore, the analysis of the sample composition supports this conclusion, as the samples differ only marginally and are broadly in line with the general population regarding age, sex, and regional characteristics. Finally, the availability of detailed sociodemographic data and the substantially larger sample size than initially assumed allow for poststratification and weighting adjustments to address potential deviations, thereby strengthening the robustness of the findings and improving their generalizability.

### Limitations

#### Selection effects and sample composition

First, the analysis of selection effects and sample composition itself is limited. It is only possible to make comparisons with the overall sample, and consequently with nonresponders, on the basis of a limited number of register-based characteristics (sex, age, BIK classification, region). Register-based records in Germany lack further information about educational attainment or occupational status. As a result, analyses of certain characteristics, such as educational bias, can only be analyzed at the aggregate level of official statistics. For the third research question, only variables that can be compared with external official benchmark data, such as the German microcensus, can be utilized.

In the analysis of sample composition, substantial deviations were observed regarding education level and German citizenship for the sample of the panel recruitment survey and the sample of registered panelists. In particular, for education, the samples differ in such a way that the proportion of lower educated individuals dropped by more than three additional percentage points. This indicates that the registration process increased the educational bias. Analysis of the registered panelists compared to the sample of the panel recruitment study shows that, besides sociodemographic charachteristics, public health-realated outcome variables also influence the willingness to participate in the panel. The analysis showed, that individuals with self-stated chronic deseases were more likely to join the panel, while those with poor self-rated subjective health were less likely to register, compared to respondents with higher self-rated subjective health. Health behavior related variables also mattered – occasional smokers were less likely to register for the panel compared to non-smokers, whereas regular physical activity substantially increased the likelihood of registration for the panel. In contrast, dietary habits and general perceived attention to health showed no effects. Taken together, these results do not reveal a clear registration pattern: on the one hand, individuals with healthier behavior and better health status were more likely to become participants in the panel. On the other hand, individuals reporting chronic disease also showed a higher likelihood of registering. The first result suggests a bias towards a healthier panel, whereas the latter indicates that less healthy individuals are overrepresented. It is noteworthy that the observed selection effects related to health characteristics are considerably smaller than those observed for sociodemographic factors such as education and German citizenship. This confirms the previous evidence that health survey respondents may not be fully representative of the general population, and shows that the impact of selection effects must be carefully assessed rather than being assumed to be negligible.

As already mentioned, this recruitment study also shows a potential education bias among lower- and higher-educated respondents [59, 64]. Above all, the underrepresentation of the low-educated population group (lower secondary school leaving certificate to no qualifications at all) poses challenges for the infrastructure in the future. Nevertheless, owing to the large sample size, it was possible to recruit such a large subsample of people with a low level of education that subgroup analyses with high statistical power can also be carried out for this group. A comparison with other studies in Germany also shows that the education bias (measured in the absolute percentage point difference from the benchmark of the official statistics) is within the expected average, with a value of nearly 11% points in the panel recruitment survey [23]. Unfortunately, this comparison also reveals that educational bias in studies in Germany has gradually increased over the last few years. There are several reasons for this. Only a few reasons could be addressed by researchers, e.g., reducing the burden of participation for people with lower education. For further refresher samples, the use of interviewers (the so-called “knock-at-door” strategy) in so-called hard-to-reach subgroups is a possible option to motivate less educated people to take part via questionnaires and hence reduce educational bias.

#### Further limitations

Another limitation relates to the panel’s current lack of multi-language capability, which may contribute to the underrepresentation of people without German citizenship, particularly those with limited German proficiency. Offering questionnaires in multiple languages could be a meaningful step toward improving accessibility for some groups; however, it is unlikely to fully resolve existing participation gaps on its own. Multilingual materials should therefore be considered as one component within a broader set of strategies aimed at enhancing the inclusion of underrepresented populations. Future extensions of the panel should explore how multilingual approaches – together with additional measures to reduce participation barriers – can support the integration of people with migration backgrounds [65]. In addition to the use of multi-language questionnaires, providing information on the study websites and offering invitation materials in various languages should also be considered to further enhance accessibility and inclusiveness.

### Conclusion and outlook

With the approach of a mixed-mode study design, which focuses on a push-to-web strategy (for the age group under 70 years), one of the largest probability-based mixed-mode health panels in Europe could be set up, which can be used for population-based statements. The use of an incentive scheme combining pre-paid and post-paid cash incentives may have contributed to a comparatively high response and recruitment rate when compared to other similar studies using no monetary incentives. This hypothesis is supported by a comparison with very similar mixed mode studies by the RKI. For instance, the response rate for GEDA 2014/15 EHIS was 27.6% (10 years prior to the recruitment study described in this field report), which was also recruited using the same selection framework and only integrated conditional non-cash incentives for a limited number of age groups [33].

The ‘Health in Germany’ panel is another example of panel studies in the context of Germany that have built up a comprehensive sample using a mixed-mode approach [66]. In this sense, this field report could serve as an example to other survey researchers working on similar study projects.

The panel data will support cross-sectional analyses within the RKI’s health monitoring program. Unlike standard cross-sectional studies, panel data are susceptible to selection biases introduced during both registration and subsequent survey wave participation. This paper provides an initial examination of the sample composition and selection biases, revealing a mix of slightly reinforcing and opposing trends between the recruitment study and panel registration. While the observed selection effects are not alarming, ongoing monitoring within the panel’s regular surveys is essential. To mitigate potential biases, sample weighting, accounting for the registration and participation processes, is necessary. A more detailed explanation of the weighting scheme, will be presented in a separate article later this year.

The panel infrastructure described here will be expanded in the next few years. After the successful implementation of regular operations for annual surveys as part of health monitoring (expansion stage I), various examinations and measurements, such as blood samples, blood pressure measurements and anthropometric surveys, are to be carried out on a partial sample (expansion stage II). A concept for the introduction of these examination modules is currently being developed. There are also plans to open the panel for self-recruitment of a non-probability-based sample. This expansion stage is planned as a complementary measure to the existing probability-based sample. The gold standard of randomly recruited panelists will not be mixed with the self-recruited non-probability panelists. The two samples will remain separate and will be used for different purposes and studies. A central element of the third expansion stage is the creation of a survey app for smartphone users. The ‘Health in Germany’ panel is going to be a central instrument for observing population health in Germany. It can be flexibly adapted to needs and serves as the basis for digital data collection. This will allow better coverage of future data needs, including in the event of a pandemic.

## Data Availability

The data from surveys conducted by the Robert Koch Institute is available free of charge to use by the scientific community as Scientific Use Files. Each Scientific Use File is comprised of the respective data record along with documentation and a description of the study, sample survey documents, code plan and user instructions. The data records can be obtained by submitting an application form through the Research Data Centre (Robert Koch Institute, MF4), which can be accessed at www.rki.de/fdz.

## Appendix

**Table 5:** Final AAPOR disposition codes distribution in panel recruitment survey

Final AAPOR disposition codes distribution in panel recruitment survey	Freq.	Percent	Cum.
1.0 Returned questionnaire			
1.1 Complete	62,556	37.49	37.49
1.2 Partial	181	0.11	37.60
2.0 Eligible, non-interview			
2.11 Refusal	787	0.47	38.07
2.112 Known-respondent Refusal (Hotline/E-Mail/Letter)	2,360	1.41	39.48
2.113 Empty questionnaire returned PAPI (Implicit Denial)	1,443	0.86	40.34
2.25 Notification that respondent was unavailable during field period	224	0.13	40.47
2.312 Reminder letter: recipient deceased [returned mail, hotline]	528	0.32	40.79
2.32 Physically or mentally unable (information via hotline or general contact)	217	0.13	40.92
2.33 Language (cannot participate due to language problems) [Hotline]	1	0.00	40.92
2.35 Reminder letter: Undeliverable/recipient cannot be determined at the specified address [returned mail]	9,627	5.77	46.69
2.36 Someone other than respondent completes questionnaire or interview and later determined ineligible	831	0.50	47.19
2.372 Reminder letter: Recipient moved [postal returns]	751	0.45	47.64
3.0 Unknown eligibility, non-interview			
3.10 Unknown (there has been no/never response from this address)	86,805	52.03	99.67
3.231 Invitation letter: Acceptance refused [returned mail -> postmark]	92	0.06	99.73
3.31 Invitation letter: Undeliverable/recipient cannot be determined at the specified address [returned mail]	358	0.21	99.94
4.0 Not eligible, returned			
4.1 Invitation letter (on first contact): Recipient moved [returned mail]	2	0.00	99.94
4.9 Invitation letter: Recipient deceased	80	0.05	100.00
Total	166,843	100.00	

**Table 6:** Logistic regression models Log-Odds (Logits)

	Panel Recruitment Survey [Yes/No]			Panel registration [Yes/No]			Panel registration [Yes/No]		
Characteristic	Logit [95%-CI]	SE	p-value	Logit [95%-CI]	SE	p-value	Logit [95%-CI]	SE	p-value
Age Group									
16–29 yrs (Ref.)	REF			REF			REF		
30–39 yrs	−0.01 [−0.07, 0.05]	0.029	0.7	0.01 [−0.05, 0.07]	0.030	0.7	0.02 [−0.07, 0.11]	0.046	0.7
40–49 yrs	−0.03 [−0.09, 0.03]	0.028	0.3	−0.05 [−0.12, 0.01]	0.034	0.12	−0.12 [−0.21, −0.02]	0.047	0.014
50–59 yrs	0.05 [−0.01, 0.11]	0.030	0.10	0.01 [−0.05, 0.07]	0.029	0.7	−0.20 [−0.29, −0.12]	0.045	< 0.001
60–69 yrs	0.18 [0.11, 0.25]	0.035	< 0.001	0.11 [0.04, 0.18]	0.037	0.003	−0.27 [−0.35, −0.18]	0.043	< 0.001
70 + yrs	0.15 [0.09, 0.20]	0.030	< 0.001	−0.09 [−0.15, −0.03]	0.030	0.002	−0.56 [−0.65, −0.47]	0.045	< 0.001
Sex									
Male (Ref.)	REF			REF			REF		
Female	0.17 [0.15, 0.20]	0.014	< 0.001	0.20 [0.17, 0.22]	0.015	< 0.001	−0.01 [−0.06, 0.04]	0.026	0.7
BIK									
< 20,000 inhabitants (Ref.)	REF			REF			REF		
20,000 to < 50,000 inhabitants OR Surroundings of 50,000 to < 500,000 inhabitants	0.02 [−0.05, 0.10]	0.039	0.5	0.05 [−0.03, 0.12]	0.039	0.2	0.04 [−0.05, 0.14]	0.048	0.4
Core city 50,000 to < 500,000 inhabitants OR Surroundings of 500,000 + inhabitants	−0.04 [−0.13, 0.04]	0.042	0.3	0.01 [−0.07, 0.10]	0.043	0.8	0.10 [0.00, 0.21]	0.053	0.053
Core city 500,000 + inhabitants	−0.07 [−0.15, 0.01]	0.039	0.072	0.00 [−0.08, 0.08]	0.040	> 0.9	0.12 [0.03, 0.22]	0.050	0.013
Region									
Northeast (Ref.)	REF			REF			REF		
Northwest	0.10 [0.00, 0.20]	0.050	0.041	0.11 [0.00, 0.22]	0.054	0.043	0.10 [0.02, 0.19]	0.045	0.022
Central-East	0.07 [−0.07, 0.21]	0.070	0.3	0.02 [−0.11, 0.16]	0.070	0.7	−0.08 [−0.16, 0.01]	0.045	0.091
Central-West	0.03 [−0.05, 0.11]	0.043	0.5	0.02 [−0.07, 0.12]	0.048	0.6	0.12 [0.04, 0.20]	0.039	0.002
South	0.05 [−0.03, 0.14]	0.044	0.2	0.05 [−0.04, 0.14]	0.047	0.3	0.09 [0.00, 0.18]	0.044	0.040
Education									
Low (Ref.)							REF		
Medium							0.44 [0.38, 0.50]	0.030	< 0.001
High							0.71 [0.64, 0.78]	0.035	< 0.001
German citizenship									
No (Ref.)							REF		
Yes							0.72 [0.63, 0.81]	0.046	< 0.001
Household size									
Single-person household (Ref.)							REF		
Multi-person household							0.09 [0.03, 0.14]	0.028	0.002
BMI									
Normal weight (18.5 < = BMI < 25) (Ref.)							REF		
Underweight (BMI < 18.5)							0.05 [−0.10, 0.20]	0.077	0.5
Overweight (25 < = BMI < 30)							0.01 [−0.05, 0.06]	0.028	0.8
Obesity (BMI > = 30)							0.07 [0.01, 0.14]	0.032	0.025
Self-rated health									
Very good/good/fair (Ref.)							REF		
Bad/very bad							−0.19 [−0.28, −0.09]	0.049	< 0.001
Self-rated mental health									
Excellent/very good/good (Ref.)							REF		
fair/poor							0.05 [−0.01, 0.11]	0.033	0.13
Paying Attention to Health									
Not at all/less strong/moderate (Ref.)							REF		
Strong/very strong							0.03 [−0.02, 0.09]	0.027	0.2
Satisfaction: Life in general									
Scale value 1 to 3 (Ref.)							REF		
Scale value 4 to 7							0.03 [−0.06, 0.12]	0.046	0.5
Scale value 8 to 10							0.08 [−0.01, 0.18]	0.047	0.074
Red meat									
Never (Ref.)							REF		
Daily or several times a day							−0.32 [−0.52, −0.13]	0.100	0.001
4 to 6 times per week							−0.13 [−0.27, 0.00]	0.069	0.053
1 to 3 times per week							−0.06 [−0.19, 0.06]	0.064	0.3
Less than once per week							0.02 [−0.10, 0.14]	0.061	0.7
Sausage Products									
Never (Ref.)							REF		
Daily or several times a day							0.08 [−0.05, 0.22]	0.067	0.2
4 to 6 times per week							0.13 [0.00, 0.26]	0.065	0.050
1 to 3 times per week							0.03 [−0.08, 0.14]	0.056	0.6
Less than once per week							0.04 [−0.06, 0.14]	0.053	0.4
Smoking									
Non-smoker (Ref.)							REF		
Daily smoking							−0.04 [−0.10, 0.02]	0.032	0.2
Occasional smoking							−0.16 [−0.26, −0.06]	0.053	0.003
Chronic Disease									
No (Ref.)							REF		
Yes							0.26 [0.21, 0.31]	0.025	< 0.001
Sport									
No sporting activities (Ref.)							REF		
Less than 1 h per week							0.13 [0.05, 0.20]	0.038	< 0.001
1 to less than 2 h per week							0.19 [0.12, 0.26]	0.034	< 0.001
2 to less than 4 h per week							0.28 [0.20, 0.36]	0.039	< 0.001
4 h per week and more							0.29 [0.21, 0.38]	0.045	< 0.001
Health risk due to climate change									
Scale value 1 to 3 (Ref.)							REF		
Scale value 4 to 7							0.07 [0.02, 0.12]	0.026	0.006
Scale value 8 to 10							0.07 [0.00, 0.15]	0.036	0.039
Waited for appointment									
No (Ref.)							REF		
Yes							−0.06 [−0.11, −0.01]	0.026	0.021
No need for examination or treatment							−0.05 [−0.13, 0.02]	0.039	0.2
N.Obs.	166,843			166,843			60,632		
AIC	219,776			199,659			62,883		

**Table 7:** Logistic regression models AME

	Panel Recruitment Survey [Yes/No]			Panel registration [Yes/No]			Panel registration [Yes/No]		
Characteristic	AME [95%-CI]	SE	p-value	AME [95%-CI]	SE	p-value	AME [95%-CI]	SE	p-value
Age Group									
16–29 yrs	REF			REF			REF		
30–39 yrs	−0.003 [−0.016,0.01]	0.0067	0.675	0.003 [−0.01,0.015]	0.0063	0.664	0.003 [−0.011,0.016]	0.0069	0.689
40–49 yrs	−0.007 [−0.019,0.006]	0.0064	0.290	−0.01 [−0.024,0.003]	0.0068	0.125	−0.018 [−0.033,−0.004]	0.0075	0.014
50–59 yrs	0.011 [−0.002,0.025]	0.007	0.104	0.002 [−0.01,0.014]	0.006	0.711	−0.033 [−0.047,−0.019]	0.0071	< 0.001
60–69 yrs	0.042 [0.026,0.058]	0.0082	< 0.001	0.023 [0.008,0.038]	0.0077	0.003	−0.044 [−0.058,−0.03]	0.007	< 0.001
70 + yrs	0.034 [0.021,0.048]	0.0071	< 0.001	−0.019 [−0.03,−0.007]	0.006	0.002	−0.099 [−0.114,−0.084]	0.0078	< 0.001
Sex									
Male	REF			REF			REF		
Female	0.041 [0.035,0.048]	0.0033	< 0.001	0.04 [0.034,0.046]	0.0031	< 0.001	−0.002 [−0.01,0.007]	0.0044	0.688
BIK									
< 20,000 inhabitants	REF			REF			REF		
20,000 to < 50,000 inhabitants OR Surroundings of 50,000 to < 500,000 inhabitants	0.006 [−0.012,0.024]	0.0091	0.517	0.01 [−0.006,0.025]	0.008	0.224	0.007 [−0.009,0.024]	0.0084	0.393
Core city 50,000 to < 500,000 inhabitants OR Surroundings of 500,000 + inhabitants	−0.01 [−0.03,0.009]	0.0099	0.301	0.003 [−0.014,0.02]	0.0087	0.759	0.018 [0,0.036]	0.0092	0.054
Core city 500,000 + inhabitants	−0.017 [−0.035,0.001]	0.0092	0.066	0.001 [−0.015,0.017]	0.0082	0.933	0.021 [0.005,0.038]	0.0086	0.013
Region									
Northeast	REF			REF			REF		
Northwest	0.024 [0.001,0.047]	0.0115	0.039	0.023 [0.001,0.044]	0.011	0.040	0.018 [0.003,0.033]	0.0077	0.021
Central-East	0.015 [−0.016,0.046]	0.0158	0.336	0.004 [−0.023,0.031]	0.0138	0.777	−0.014 [−0.029,0.002]	0.008	0.090
Central-West	0.007 [−0.012,0.026]	0.0098	0.495	0.005 [−0.014,0.024]	0.0097	0.626	0.02 [0.007,0.034]	0.0067	0.002
South	0.012 [−0.008,0.031]	0.0099	0.236	0.011 [−0.008,0.029]	0.0095	0.267	0.015 [0.001,0.03]	0.0075	0.040
Education									
Low							REF		
Medium							0.082 [0.072,0.094]	0.0058	< 0.001
High							0.124 [0.113,0.137]	0.0062	< 0.001
German citizenship									
No							REF		
Yes							0.139 [0.12,0.159]	0.0099	< 0.001
Household size									
Single-person household							REF		
Multi-person household							0.015 [0.006,0.025]	0.0048	0.002
BMI									
Normal weight (18.5 < = BMI < 25)							REF		
Underweight (BMI < 18.5)							0.009 [−0.016,0.034]	0.0128	0.487
Overweight (25 < = BMI < 30)							0.001 [−0.008,0.011]	0.0048	0.802
Obesity (BMI > = 30)							0.012 [0.002,0.023]	0.0054	0.023
Self-rated health									
Very good/good/fair							REF		
Bad/very bad							−0.033 [−0.051,−0.015]	0.0089	< 0.001
Self-rated mental health									
Excellent/very good/good							REF		
fair/poor							0.008 [−0.002,0.019]	0.0054	0.126
Paying attention to health									
Not at all/less strong/moderate							REF		
Strong/very strong							0.006 [−0.003,0.014]	0.0046	0.225
Satisfaction: Life in general									
Scale value 1 to 3							REF		
Scale value 4 to 7							0.006 [−0.01,0.022]	0.008	0.460
Scale value 8 to 10							0.014 [−0.001,0.03]	0.008	0.077
Red meat									
Never							REF		
Daily or several times a day							−0.057 [−0.093,−0.022]	0.0181	0.002
4 to 6 times per week							−0.023 [−0.045,0]	0.0115	0.049
1 to 3 times per week							−0.011 [−0.032,0.01]	0.0106	0.306
Less than once per week							0.003 [−0.016,0.023]	0.01	0.735
Sausage products									
Never							REF		
Daily or several times a day							0.014 [−0.008,0.037]	0.0114	0.216
4 to 6 times per week							0.021 [0,0.043]	0.011	0.052
1 to 3 times per week							0.005 [−0.014,0.024]	0.0097	0.614
Less than once per week							0.007 [−0.011,0.025]	0.0092	0.447
Smoking									
Non-smoker							REF		
Daily smoking							−0.007 [−0.018,0.004]	0.0055	0.215
Occasional smoking							−0.028 [−0.047,−0.009]	0.0095	0.004
Chronic diseases									
No							REF		
Yes							0.044 [0.036,0.052]	0.0042	< 0.001
Sport									
No sporting activities							REF		
Less than 1 h per week							0.023 [0.01,0.036]	0.0067	< 0.001
1 to less than 2 h per week							0.034 [0.022,0.046]	0.006	< 0.001
2 to less than 4 h per week							0.048 [0.035,0.061]	0.0067	< 0.001
4 h per week and more							0.051 [0.036,0.066]	0.0076	< 0.001
Health risk due to climate change									
Scale value 1 to 3							REF		
Scale value 4 to 7							0.012 [0.004,0.021]	0.0045	0.006
Scale value 8 to 10							0.013 [0.001,0.025]	0.0061	0.037
Waited for appointment									
No							REF		
Yes							−0.01 [−0.019,−0.002]	0.0044	0.021
No need for examination or treatment							−0.009 [−0.022,0.004]	0.0067	0.172

**Table 8:** Chi-square tests (X2) and effect sizes (Cramér’s V) comparing sample distributions with benchmark distributions

Characteristic	Gross^1^ [%]	RS^1^ [%]	Panel^1^ [%]	RS^1^ Weighted^2^ [%]	Panel^1^ Weighted^2^ [%]
Age					
X^2^	597.24	328.34	131.32	23.97	72.9
p-value	< 0.001	< 0.001	< 0.001	< 0.001	< 0.001
Cramér’s V	0.042	0.051	0.037	0	0.001
BIK Classification					
X^2^	913.93	226.86	235.68	0.14	0.15
p	< 0.001	< 0.001	< 0.001	0.998	0.997
Cramér’s V	0.052	0.042	0.05	0	0
Education^3^					
X^2^	2198.28	2199.36	2799.27	354.72	766
p-value	< 0.001	< 0.001	< 0.001	< 0.001	< 0.001
Cramér’s V	0.133	0.133	0.172	0.002	0.003
Federal State					
X^2^	19007.72	6976.31	5351.61	< 0.05	0.15
p-value	< 0.001	< 0.001	< 0.001	1	1
Cramér’s V	0.239	0.235	0.237	0	0
German Citizenship					
X^2^	2380.33	2381.28	2374.08	432.48	756.28
p-value	< 0.001	< 0.001	< 0.001	< 0.001	< 0.001
Cramér’s V	0.138	0.138	0.158	0.002	0.004
Household Size					
X^2^	138.79	138.72	182.05	67.57	84.32
p-value	< 0.001	< 0.001	< 0.001	< 0.001	< 0.001
Cramér’s V	0.033	0.033	0.044	0.001	0.001
Sex					
X^2^	7.52	42.43	43.62	16.65	19.64
p-value	0.006	< 0.001	< 0.001	< 0.001	< 0.001
Cramér’s V	0.005	0.018	0.021	0	0.001

p-value (Chi-square test)

^1^Gross= Gross sample; RS = Recruitment Panel Survey; Panel = Registered Panelists;

^2^Weighting accounts for different selection probabilities from sampling design

^3^Casmin classification (Comparative Analyses of Social Mobility in Industrial Nations [46])

## Abbreviations

RKI: Robert Koch Institute
KiGGS: German Health Interview and Examination Survey for Children and Adolescents (“Studie zur Gesundheit von Kindern und Jugendlichen in Deutschland“)
DEGS: German Health Interview and Examination Survey for Adults (“Studie zur Gesundheit Erwachsener in Deutschland“)
GEDA: German Health Update (“Gesundheit in Deutschland aktuell“)
GESIS: Leibniz Institute for the Social Sciences (“Leibniz-Institut für Sozialwissenschaften“)
SOEP: Socio-Economic Panel at the German Institute for Economic Research, DIW (“Deutsches Institut für Wirtschaftsforschung”)
EMA: residents’ registration offices (“Einwohnermeldeämter”)
EHIS: European Health Interview Survey
CAWI: Computer-Assisted Web Interviewing
PAPI: PaperandPencil Interviewing
AAPOR: American Association for Public Opinion Research
RR1: Response Rate 1
RECR: Recruitment Rate
AME: Average marginal effect
